# Influence of Process Parameter and Build Rate Variations on Defect Formation in Laser Powder Bed Fusion SS316L

**DOI:** 10.3390/ma18020435

**Published:** 2025-01-18

**Authors:** Tasrif Ul Anwar, Patrick Merighe, Rahul Reddy Kancharla, Boopathy Kombaiah, Nadia Kouraytem

**Affiliations:** 1 Mechanical and Aerospace Engineering Department, Utah State University, Logan, UT 84322-4130, USA; 2 Post-Irradiation Examination Department, Materials and Fuels Complex, Idaho National Laboratory, Idaho Falls, ID 83415-2209, USA

**Keywords:** laser powder bed fusion, X-ray computed tomography, porosity defects, build rate

## Abstract

Laser powder bed fusion (LPBF) is an additive manufacturing process that has gained interest for its material fabrication due to multiple advantages, such as the ability to print parts with small feature sizes, good mechanical properties, reduced material waste, etc. However, variations in the key process parameters in LPBF may result in the instantiation of porosity defects and variation in build rate. Particularly, volumetric energy density (VED) is a variable that encapsulates a number of those parameters and represents the amount of energy input from the laser source to the feedstock. VED has been traditionally used to inform the quality of the printed part but different values of VED are presented as optimal values for certain material systems. An optimal VED value can be maintained by changing the key process parameters so that various combinations yield a constant value. In this study, an optimal constant VED value is maintained while printing SS316L with variable key processing parameters. Porosity analysis is performed using optical microscopy, as well as X-ray computed tomography, to reveal the volume density and distribution of those pores. Two primary defect categories are identified, namely lack of fusion and porosity induced by balling defects. The findings indicate that, even at optimal VED, variations in process parameters can significantly influence defect type, underscoring the sensitivity of defect formation to the variation of these parameters. Furthermore, a minor change in the build rate, driven by adjustments in process parameters, was found to influence defect categories. These findings emphasize that fine tuning the process parameters and build rate is essential to minimize defects. Finally, fiducial marks have been identified as a source of unintentional porosity defects. These results enable the refinement of process parameters, ultimately optimizing LPBF to achieve enhanced material density and expedite the printing.

## 1. Introduction

Additive manufacturing (AM) is an effective manufacturing process for complex engineering parts when the cost of fabrication using traditional methods is quite high [1]. AM can decrease fabrication lead time, minimize the weight of manufactured components, and reduce waste generation. In the large domains of AM processes, laser powder bed fusion (LPBF) stands out due to its distinctive abilities such as facilitating the production of complex geometries, exhibiting enhanced precision, handling a wide range of materials, etc. [2,3]. In the LPBF process, the material is printed layer by layer with the help of a laser beam to fabricate three-dimensional objects based on computer-aided design. The printing process is performed in three steps: (1) Initially, homogeneous powders are spread to the build plate by maintaining a layer thickness; (2) a laser is used to melt these powders to form the desired shape; and (3) the build plate is lowered and fresh powders are distributed evenly to the build plate. These processes continue until the full three-dimensional part is fabricated [4].

Despite the recent growth of AM techniques, there remains a lack of comprehensive understanding of the relation between the process, structure, and properties in LPBF as opposed to conventionally manufactured components [5]. Variation in the build quality may occur primarily as a result of employing different laser parameters [6], scan parameters [7], particle size distribution [8], etc. The presence of these variances might lead to the initiation of defects in the printed components, which can have a substantial impact on their mechanical properties. The most common process-related defects in LPBF include lack of fusion (LOF), keyhole (K), and balling [2,9]. These defect types will be briefly outlined in the following paragraphs.

A lack of bonding between the melt pools and layers results in a lack of fusion (LOF) porosity [10]. Insufficient laser energy input can result in fusion deficits, leading to incomplete melting of materials. Moreover, if the powder receives an adequate amount of energy to undergo melting, yet the resulting melt pools fail to overlap with adjacent ones during the printing process, it will also lead to the formation of LOF defects [11]. Research has revealed that the issue of LOF persists even when employing techniques like hot isostatic pressing, and subsequent heat treatment has no impact on the morphology of interconnected pores [12]. Investigations have also demonstrated that the presence of LOF defects can diminish the fracture toughness of 3D-printed components depending on factors like the build orientation [13]. Furthermore, LOF defects have been associated with limited elongations of fracture surfaces in processes such as laser metal wire deposition [14], a technique that uses a metal wire as the feedstock instead of powders melted by a laser heat source.

A high laser power and a low laser velocity may lead to the formation of keyhole porosity. At those process parameters, the emergence of metallic vapor results in the formation of recoil pressure on the melt pool, leading to a keyhole-shaped melt pool creation. When the melt pool loses stability, it collapses and pinches off gas bubbles from the vapor depression, leading to the formation of a keyhole defect [15]. Studies have demonstrated that the addition of nanoparticles can effectively prevent the collapse and fluctuation of keyholes [16]. Presently, researchers are employing high-speed synchrotron X-ray imaging to comprehensively understand the dynamics of keyholes. In their research, Kouraytem et al. [17] examined the dynamics of the keyhole and observed that, as the laser velocity increases while keeping the laser power constant, there is a decrease in the fluctuation of the keyhole. Cunningham et al. [18] also investigated the vapor depression and revealed a correlation between the depth of the keyhole and the angle of its front wall, indicating that when the front wall angle is greater than 77°, keyhole pores arise in the print [2].

The phenomenon known as balling is characterized by the breaking up of the melt track prior to its solidification [19]. This kind of flaw manifests in the shape of a hump or bead when welding at a faster velocity with a greater current, which, in AM, is analogous to higher laser scanning speed and power [20]. Current research suggests diverse mechanisms for balling formation, three of which are discussed herein [2,21,22]. In the first mechanism, the formation of balls is attributed to inadequate wettability and elevated viscosity, which result from poor fusion of the powder particles. As the laser scans, the presence of both solid and liquid phases is enhanced, which subsequently gives rise to the generation of disconnected balls [2]. In the second mechanism, Plateau–Rayleigh instability is identified as a contributing factor to balling. Significant variations in surface tension within the molten pool disrupt the continuity of the molten track, causing it to break into smaller segments, forming balls [21]. In the last mechanism, oxygen content during printing has been found as a significant factor in the ball formation as its presence facilitates the production of balls during the printing procedure [22].

It has been observed that the type and characteristics of the defects that develop are significantly influenced by the key processing parameters (i.e., laser power, scan speed, layer thickness, and hatch spacing), which are directly related to the volumetric energy density (VED) [2]. VED is the amount of energy delivered to the build per unit volume during the printing process [23]. As VED plays an important role in determining defect types, it is important to develop a better understanding of it. Previous research findings indicate that an increase in energy density leads to the occurrence of keyhole defects, whereas a decrease will result in the lack of fusion defects [24,25].

In this research, SS316L was chosen due to its enhanced corrosion resistance and high strength at increased temperatures, which makes it useful in fields such as the marine, biomedical, and energy generation industries [26]. Though VED does not necessarily ensure the highest material density, a range of VED values can be identified to achieve higher-density materials [27]. Several studies have provided recommendations on the energy density for LPBF SS316L, which have yielded dense samples. In a study conducted by Cherry et al. [28], it was observed that an energy density of 104.52 J/mm^3^ resulted in the lowest observed porosity at 0.38% when printed in a Renishaw AM 250 printer (Renishaw, Wotton-under-Edge, UK). Another study by Tucho et al. [26] reported achieving the lowest porosity in the SS316L samples when the VED was maintained at 80 J/mm^3^ in a SLM 280HL machine (SLM Solutions Group AG, Lübeck, Germany). According to Eliasu et al. [29], the LPBF samples exhibited the greatest densification in an EOS M280 printer (EOS GmbH, Krailing, Germany) when the VED was maintained at 88.89 J/mm^3^. The analysis by Vallejo et al. [30] suggested that samples printed in an SLM 125HL printer (SLM Solutions Group AG, Lübeck, Germany) with VED in the range of 46–127 J/mm^3^ resulted in a maximum part density of 99.8%. These studies suggest that a range of 80–105 J/mm^3^ can lead to the greatest densification in SS316L depending on the machine type.

Previous research exploring the VED parameter has aimed to produce components with high material density. It is important to note that an optimal VED can be achieved by adjusting the process parameters in such a way that different combinations of process parameters result in the same VED. This approach can lead to variations in the types of defects observed. Additionally, adjusting the process parameters while maintaining an optimal VED allows for variations in the build rate of the printed parts. A higher build rate reduces production time, minimizing delays. This study hypothesizes that systematically altering key laser process parameters while keeping an optimal VED will reveal variations in build rates and defect types. These insights can be leveraged to refine the process parameters, ultimately optimizing LPBF for enhanced material density and faster printing.

## 2. Materials and Methods

### 2.1. Fabrication Process

SS316L samples were fabricated using Praxair gas-atomized powder that had a particle size distribution ranging from 15–45 μm [31]. The printing procedure was conducted using a Concept Laser M2 printer (GE Additive, Bayern, Germany). Throughout the printing procedure, the preheat temperature was maintained at a constant value of 25 °C, while argon gas was employed as an inert medium. Based on literature findings [26,28,29,30], all of the samples were printed with a VED value of 100 J/mm^3^, and they were selected as a middle value of the range, which was then investigated by the research community as they are expected to yield the highest relative density. While other studies have reported VED values such as 80 J/mm^3^ [26], 104.52 J/mm^3^ [28], 88.89 J/mm^3^ [29] and 46–127 J/mm^3^ [30] for maximizing density, directly comparing these results with our research is challenging due to the limited details on the laser parameters in the existing literature [26,28,29,30]. When comparing the laser beam diameters in this study with the literature, the types of lasers used in each study were different. Cherry et al. [28] used a YAG laser with a $d_{0}$ = 70 μm spot diameter; Eliasu et al. [29] did not mention the laser type but included the spot size of 100 μm; Vallejo et al. [30] used an IPG fiber laser with a beam diameter of $d_{0}$ = 70 μm; and Tucho et al. [26] mentioned a fiber laser was used but no spot size was mentioned. This variability in laser types and beam diameters hinders a direct comparison between the current study and the studies in the literature.

The VED of the printed samples was consistently maintained at the same value by utilizing Equation (1) to adjust the laser key process parameters.

(1) $$VED=\frac{P}{V\times H\times L}$$

where *P* (W) is the laser power, *V* (mm/s) is the laser scan speed, *H* (μm) is the hatch spacing, and *L* (μm) is the layer thickness. A total of 13 distinct cylindrical samples of 20 mm in height and 10 mm in diameter were fabricated by manipulating the laser power and velocity to relate the mechanisms underlying the development of porosity defects. The build rate of these samples was calculated using the following Equation (2):

(2) BuildRate=V×H×L

The detailed printing process parameters for the samples are in Table 1. During this printing process, the layer thickness, hatch spacing, and laser spot diameter were kept constant at *L* = 50 μm, *H* = 70 μm, and $d_{0}$ = 125 μm, respectively.

**Figure 1:**
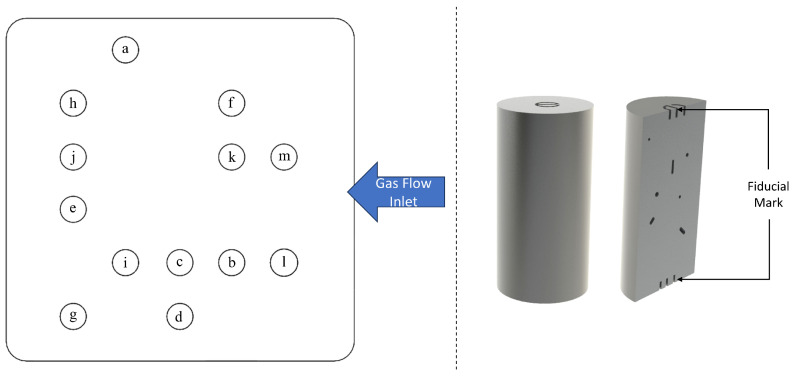
(**Left**) Specimen layout on the build platform; (**Right**) cylindrical sample with fiducial marking.

To aid in the identification of the samples, fiducial marks in the form of different numbers were debossed into both the top and bottom layers of the printed parts (Figure 1 (Right)) during the fabrication process. These markers, each measuring 1 mm in height, were embedded directly into the material structure. This marking system ensured easy identification and correct orientation of the samples after fabrication, allowing for consistency during characterization and analysis.

Following the completion of the printing, the specimens were extracted from the plate via wire electrical discharge machining (EDM). The samples were then cut along their long edges using EDM, and the half-cylinder samples were polished to conduct further analysis using optical microscopy (OM) and X-ray computed tomography (XCT), as described next.

### 2.2. X-Ray Computed Tomography

High-energy electromagnetic radiation, known as X-rays, was employed to investigate the internal defect structure of the half cylinders. X-ray computed tomography (XCT) is a non-destructive method that enables examination of both the internal and external geometrical characteristics of a sample [32]. The scan of the samples was performed using a Nikon XTH 225 (Nikon Metrology, Leuven, Belgium) and a ZEISS Xradia 520 Versa scanner (Carl Zeiss AG, Oberkochen, Germany) at the micro-CT facilities of Utah State University and Idaho National Lab, respectively. The LPBF half-cylinders were initially mounted in a sample holder to mitigate any potential sample displacement during the scanning process. The samples were scanned at a slight angle to reduce the impact of edge artifacts. During the scanning procedure, the effective pixel size of the XCT was adjusted to 9 μm. This adjustment was made while modifying the beam energy, beam current, and power to achieve optimal X-ray penetration.

In the present analysis, the X-ray beam energy was maintained within the range of 135–185 kV, while an exposure duration of 1 s was employed. To decrease the impact of beam hardening, a copper filter with a thickness of 0.5 mm was used, effectively preventing background saturation by eliminating the low-energy X-rays. The scans were conducted using a circular 360-degree mode, with careful consideration given to optimizing the number of projections. Upon completion of the scanning process, the acquired sample data were reconstructed using CT-Pro software (Nikon Metrology, Leuven, Belgium) to obtain a three-dimensional volume. Three-dimensional X-ray data were used to facilitate the analyses of pores and the quantification of their dimensions and morphology.

### 2.3. Data Analysis

Object Research Systems (ORS) Inc.’s Dragonfly 2020.2 (Comet Technologies, Montreal, QC, Canada) was used to perform the statistical analysis of the XCT data [33]. Initially, the data were cropped at the upper and lower ends to eliminate the fiducial marking layers in the analysis of the samples. The data were subsequently divided into two distinct categories, namely pores and metals. To refine the analysis, morphological operations were applied to remove noise. Additionally, to minimize the influence of noise, pores with a volume of less than eight voxels were excluded. Finally, a connected component algorithm with 26 connectivity was used to separate the single largest connected object.

The software uses the segmentation technique to determine the distribution of porosity and volume density. Volume percent density and pore Feret diameter data were obtained by this process. These metrics were evaluated to understand the influence of process parameter variation on the defect formation at optimal VED and will provide valuable insights for the fracture mechanics and modeling community. The measurement of pore Feret diameter involved determining the maximum and minimum caliper distances between two points on the pore, which were selected at an arbitrary angle.

## 3. Results

### 3.1. Process Parameter Influence on Defect

Process parameters have a direct influence on the defect generated while printing. During the inspection of the printed parts via OM and XCT, it was observed that two primary categories of defects manifested in the printed specimens. The first category was lack of fusion (LOF), and the second category was balling. Between the LOF and balling process parameters, there exists an optimal process window where the formation of dense or near-dense samples can be seen. The following sections will provide a more detailed analysis of these defect categories to determine the mechanisms through which they occur.

#### 3.1.1. Lack of Fusion

**Figure 2:**
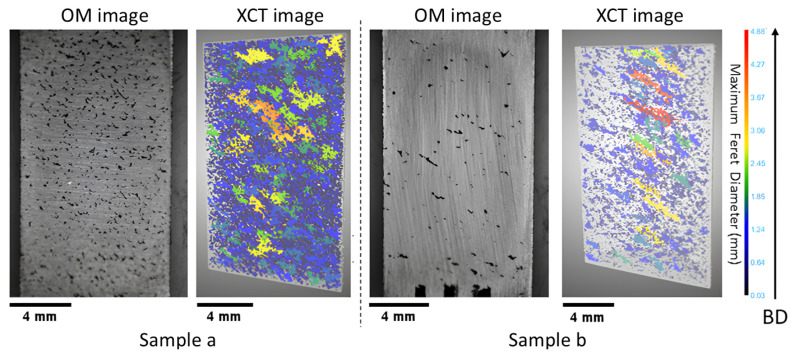
Optical microscopy (OM) image of the polished cross-sections and X-ray computed tomography (XCT) reconstructions of lack of fusion (LOF) porosity: gray represents the bulk material, and the pore Feret diameter is indicated by colors; the scale bar corresponds to 4 mm, representing the sample size; the color bar represents the maximum Feret diameter of the pores; and the arrow represents the build direction (BD).

**Figure 3:**
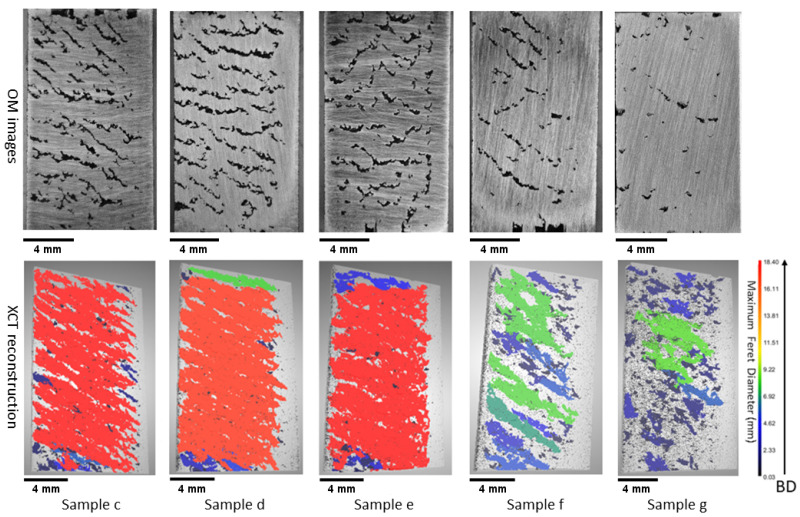
(**Top**): OM images of the polished cross-sections from the five low power–velocity parameter samples, displaying elongated irregular pores. (**Bottom**): XCT reconstruction of these five samples, showing the bulk material in gray, with pores highlighted in various colors based on their relative Feret diameters following the color bar. The scale bar corresponds to 4 mm, representing the sample size; the color bar represents the maximum Feret diameter of the pores; and the arrow represents the build direction (BD).

From the OM images of the printed parts (see Figure 2 and Figure 3), the occurrence of LOF defects was observed even when the VED was maintained at 100 J/mm^3^. These defects can include a variety of pore morphologies, ranging from elongated voids of significant length to smaller voids with irregular shapes [34]. The LOF defect criterion has been explained in different studies as a relation established between hatch spacing (*H*), layer thickness (*L*), melt pool width (*W*), and melt pool depth (*D*). Mukherjee et al. [35] suggested that sufficient melting between the layers can be identified using a non-dimensional LF index (Equation (3)), which is the ratio between the melt pool depth and layer thickness.

(3) $$LF\;index=\frac{D}{L}$$

where the LF index is a LOF identification parameter. The article proposed that, to obtain good interlayer bonding in SS316, the LF index needs to be around 1.15. Thus, if the LF index calculated value is below 1.15, it can be inferred that the penetration depth will not exceed the layer thickness, leading to the formation of LOF porosity. However, Tang et al. [11] proposed a different model (Equation (4)), suggesting that the overlap between the hatch spacing to melt pool width and layer thickness to melt pool depth must be maintained to obtain full melting in the sample to avoid LOF. The requirement to avoid LOF is calculated using a simplified inequality as follows:

(4) $${\left(\frac{H}{W}\right)}^{2}+{\left(\frac{L}{D}\right)}^{2}\leq 1$$

In this research, given the absence of in situ measurements conducted on the melt pool in SS316L, it becomes necessary to derive the width (*W*) and depth (*D*) values from the Rosenthal model [36]. In this model, the main assumption is that the melt pool exhibits a semi-circular morphology and operates in conduction mode [37], with the diameter of the melt pool being double its depth. Using the properties of the powder and laser, we can calculate the melt pool width *W* using the following equation:

(5) $$W=\sqrt{\frac{8\;\eta \;P\;}{\pi e\rho CV(T_{f}-T_{o})}}$$

where η is the absorptivity, ρ is the material density, *C* is the heat capacity, $T_{f}$ is the melting point, and $T_{o}$ is the preheat temperature. Except for absorptivity, the values of the remaining parameters can be derived from either the properties of the powder or the processing parameters. Different studies have employed a variety of values for absorptivity, spanning from 0.3 to 0.4 [38,39,40]. In this study, an absorptivity value of η = 0.3 was initially adopted. The remaining parameter values used in this research were *C* = 500 J/Kg·K, $T_{f}$ = 1808.15 K, and ρ = 8000 kg/m^3^. Using these values, the melt pool dimensions calculated with the Rosenthal model yielded a mean width of 127.61 ± 0.05 μm and a mean depth of 63.81 ± 0.02 μm.

The LOF criteria were then computed using the Mukherjee and Tang models, where a value of 1.276 ± 0.00004 was obtained from the Mukherjee model (using Equation (3)), and a value of 0.915 ± 0.005 was derived from the Tang model (using Equation (4)) while utilizing the calculated melt pool data from the Rosenthal model (using Equation (5)). This illustrates that both models predicted dense samples and a lack of porosity while using the Rosenthal model melt pool geometry. However, while observing the OM images (see Figure 2: sample (a–b), Figure 3: sample (c–g)), it became evident that numerous elongated pores were present within the sample. To further investigate the applicability of the models to the LOF cases, melt pool widths were experimentally measured at the location immediately before the start of a defect for Samples a, c, and g. The reasoning behind this melt pool measurement was that these melt pools would have the least possible remelting during fabrication. The analysis revealed that the average melt pool widths in these samples at the location before a defect was less than or equal to 110 μm, which is smaller than the theoretical melt pool width that was calculated through the Rosenthal model (127.61 ± 0.05 μm).

The experimental observations suggest that a lower absorptivity value should be used to calculate the melt pool dimensions in the models. Due to the inherent difficulty in directly quantifying the absorptivity in LPBF, there is a potential for variability in the absorptivity value (η) across different samples and processing parameters. Guo et al.’s [41] research findings indicate that, despite a fixed input energy density, fluctuation in laser absorptivity can be seen due to the roles played by laser power and velocity in developing the depression zone. For the SS316L samples, if a value of 0.27 was chosen for absorptivity and the melt pool width and depth were recalculated using the Rosenthal model, the Tang model indicated the presence of LOF defects in all cases in Samples a, b, c, d, e, f, and g. Moreover, if the absorptivity value was lowered further to 0.24, both the Mukherjee and Tang models predicted LOF in the samples evaluated in this study. As such, at the location where absorptivity dropped to 0.24–0.27, the melt pool failed to achieve sufficient dimensions for adequate overlap with each other, resulting in LOF defects.

To justify the discrepancy with the models due to absorptivity, all of the samples were subjected to XCT analysis. The analysis results are shown in Figure 2 and Figure 3. The results demonstrate that Samples a, b, c, d, e, f, and g exhibit a diverse range of LOF defects, which are characterized by elongated geometries. The sample printed at the lowest power and velocity combinations (Sample a, Figure 2) suffered from sintering defects, which is an extreme case of LOF. Sintering is a process in which particles are bonded together without undergoing complete melting. The incomplete melting of the powder occurred due to insufficient power input, despite the optimal value of a VED of 100 J/mm^3^. As a result, the XCT data indicated the existence of numerous pores, with the largest one characterized by a maximum Feret diameter of 3.07 mm.

As the magnitude of power input was increased, the samples gradually acquired sufficient energy to undergo partial melting. However, due to the relatively low power level at the initial level of 70 W, this phenomenon manifested as elongated pores that are visible in the XCT images. Such defects were observed in Samples b, c, d, and e, as shown in Figure 2 and Figure 3. In these samples, a significant proportion of the total defect volume exhibited irregular geometry. As a result of this phenomenon, the samples demonstrated the least density and the greatest pore Feret diameter within all the samples. Specifically, Sample d exhibited the lowest volume density of 90.36% with the third-largest pore Feret diameter of 17.55 mm, while Sample c exhibited the largest pore, with a Feret diameter of 18.4 mm and a volume density of 92.57%. Sample b exhibited a 98.8% volume density with a 4.88 mm Feret diameter, while Sample e exhibited a 91.17% volume density with an 18.34 mm Feret diameter. It was hypothesized that the relatively higher density of Sample b within the LOF series (compared to Samples a–g) can be attributed to its proximity to the gas flow inlet compared to the outlet (Figure 1 (Left)), as the flow is reduced near the outlet. This helps to achieved a reduced beam attenuation near the inlet due to improved gas flow, thus enhancing density and aligning with findings reported in the literature [42].

As the power increases to 150 W (Sample f in Figure 3), the maximum Feret diameter decreases compared to the lower power samples (e.g., Sample e to Sample f). Sample f was characterized by a Feret diameter of 8.03 mm and a volume density of 96.61%. Similarly, Sample g (P = 170 W) exhibited a Feret diameter of 8.27 mm with a density of 97.81%, representing a shift toward the dense process window. This observation suggests that the process parameters were transitioning from the LOF to the optimal process window, which constitutes the discussion in the next section.

#### 3.1.2. Near Dense

**Figure 4:**
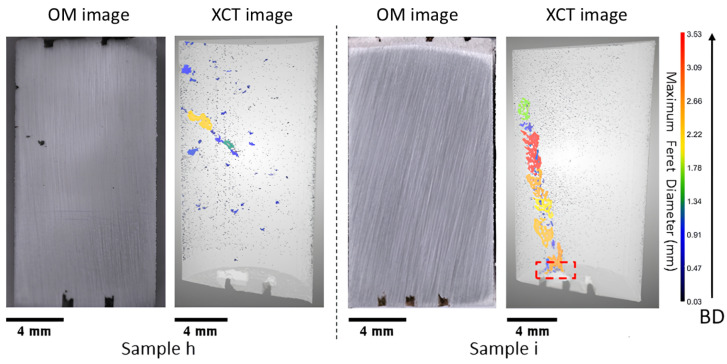
Side-by-side OM images of the polished cross-sections and XCT reconstruction: gray represents the bulk material, and the pore Feret diameter is indicated by colors; the bottom fiducial marking is shown for visualization purposes without adding the defect at that location in the porosity analysis (see discussion in Section 2.3); the scale bar corresponds to 4 mm, representing sample size; the color bar represents the maximum Feret diameter of the pores; and the arrow represents the build direction (BD).

Near-dense samples were subjected to progressively higher levels of laser power and velocity compared to the LOF samples. As the process parameter shifts in the optimal process window, dense samples are expected to be manufactured. Thus, we can observe the highest density in Samples h and i (99.88% and 99.75%, respectively). Although these samples initially looked near fully dense from the OM image (Figure 4), an additional defect was observed in these samples when observed through XCT. These defects originate either from the fiducial marking or from the inside of the sample.

In the case of Sample h (Figure 4), it displayed a substantial pore that originates from the middle of the print. We postulate that the distribution of powder was not uniform in that region, which resulted in the incomplete melting of powder particles in the root of the pore instantiation site, leading to the formation of porosity. This defect persisted intermittently for about 45 layers, appearing and disappearing in a discontinuous pattern before gradually diminishing in the sample. Sample i, on the other hand, presented an additional defect arising from the fiducial marking (see Figure 4, highlighted in a red box). It originated from the bottom layer, where the fiducial mark started and persisted intermittently throughout a significant portion of the sample but eventually diminished near the top region. We postulate that, in this case, the unevenness in the powder layer thickness evened out as more layers of powder were spread and printed.

#### 3.1.3. Balling

**Figure 5:**
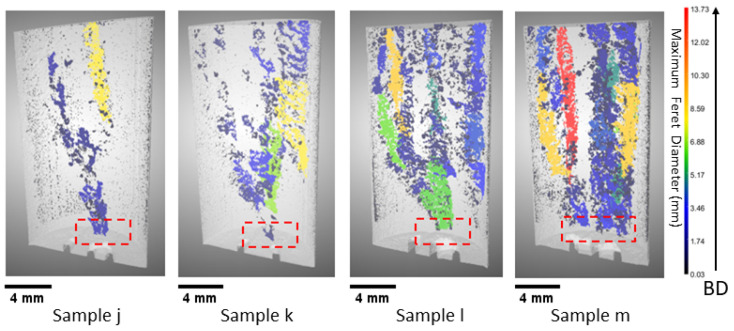
XCT 3D reconstruction of the samples processed with high power–velocity parameters leading to porosity induced by balling, as discussed in Section 3.1.3. In XCT reconstruction: gray represents the bulk material, and the pore Feret diameter is indicated by colors; the bottom fiducial mark (marked in red) indicates the suspected origin of porosity defects; the scale bar corresponds to 4 mm, representing sample size; the color bar represents the maximum Feret diameter of the pores; and the arrow represents the build direction (BD).

Based on the analysis of the XCT data, there appear to be elongated pores in the remaining samples labeled as j, k, l, and m (see Figure 5). These elongated pores are an indication of LOF. However, exhibiting LOF defects in these samples would be quite unlikely as the powders receive adequate energy to achieve complete melting, and the melt pools are expected to overlap; this is because the increase in power leads to an increase in absorptivity, resulting in a wider melt pool [43]. Furthermore, the origin of some of these pores can be traced back to the fiducial marks at the bottom layer, and their presence is consistently observed in a sporadic pattern across the build direction (see Figure 5, highlighted in red box).

When the process parameters are graphed in the P-V space, it becomes evident that these samples (j–m) fall within the high-power and high-velocity region when compared to other samples presented in this study. In this particular region, it is anticipated that either a keyhole or balling will emerge. The presence of elongated and non-spherical geometry in the pores observed in XCT precludes the occurrence of keyhole porosity formation. Thus, it is more likely that the root cause of the porosity formation is balling or beading up.

**Figure 6:**
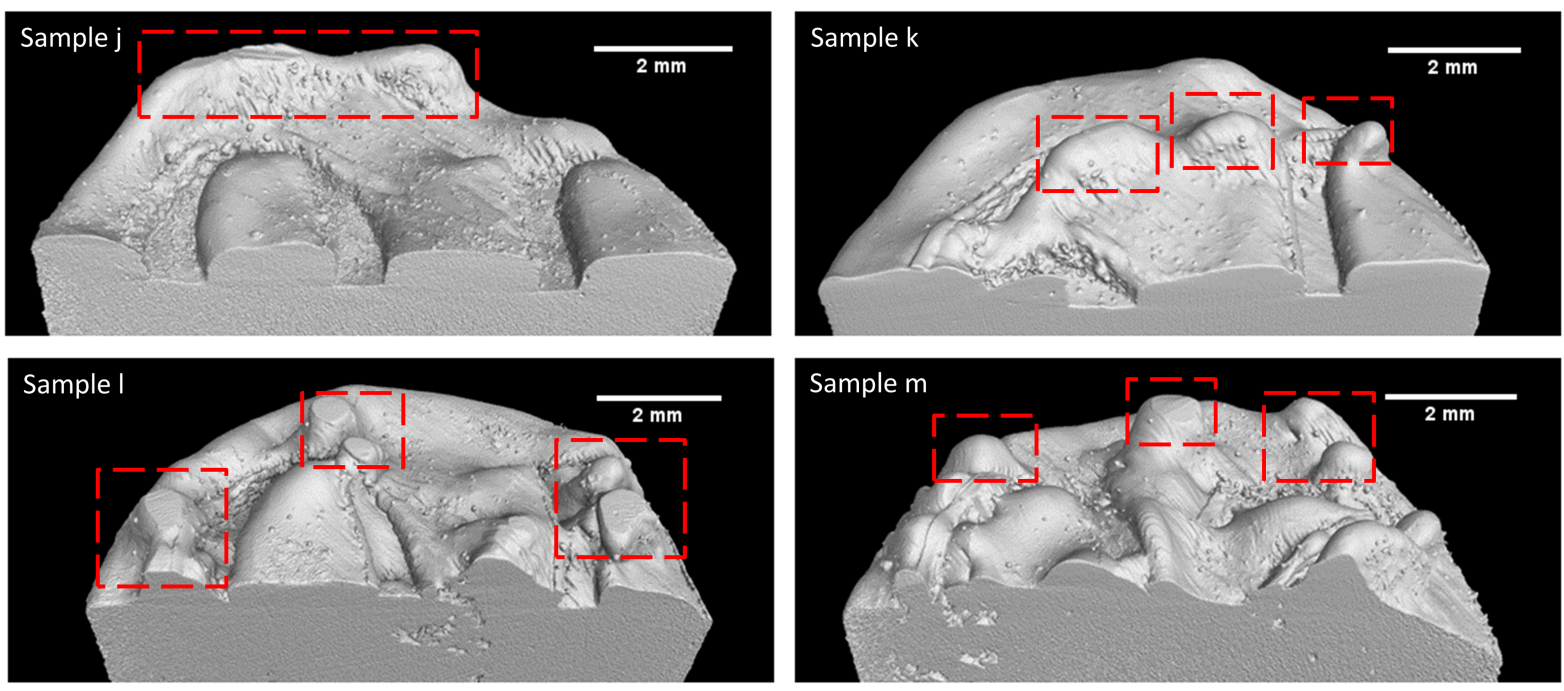
3D rendering illustrates the balling phenomenon in the samples: red box indicates balls and humps, and the scale bars correspond to 2 mm.

Li et al. [22] investigated the balling formation in the single-track LPBF of SS316L. Their research suggested that oxygen content, laser power, and scan speed play a significant role in ball formation. Based on the findings, the authors suggested that maintaining laser power at 190 W and printing speed at 600 mm/s leads to the occurrence of balling with a rough surface. Gunenthiram et al. [44] also investigated the phenomenon of balling in LPBF SS316L. The researcher’s findings indicate that balling can be attributed to insufficient wetting with the substrate, which can be observed when maintaining a laser power of 159 W and a velocity of 600 mm/s. Moreover, increasing the scanning velocity leads to the occurrence of powder spatter, hence facilitating the formation of surface roughness. The process parameters for Sample j are 230 W and 657 mm/s, which is printed with a higher power and velocity compared to the studies of Li et al. [22] and Gunenthiram et al. [44]. As such, it is more likely to undergo an increase in spatter, rough surface, and insufficient wetting during the printing of this sample. Sample j suffered from a hump, which can be easily seen in the top layer of the printed part (see Figure 6, representing an XCT reconstruction of the surface of the sample). Based on the 3D rendered image of this sample, it can be inferred that the observed sample process parameter was situated in close proximity to the dense-balling process window since there is a presence of significant humps. Thus, it is reasonable to anticipate the occurrence of balling defects in the remaining samples of k, l, and m as a result of increased power and velocity given that their respective processing parameters are in the high-power and high-velocity region.

During the solidification process of these samples, humps or beads form along the melt track. Therefore, alterations in the layer thickness are expected to give rise to defects in fusion, which are caused by balling. Among the samples, Sample j exhibited the maximum density in the balling series, measuring 99.63%, while Samples k, l, and m had densities of 99.38%, 98.41%, and 98.06%, respectively. In addition, the maximum pore Feret diameter gradually increased from 8.09 mm for j and to 13.73 mm for m. These values suggest that the percent volume density of the samples decreases and the pore Feret diameter increases with increases in power and velocity. Based on the available evidence, it is possible to identify the observed flaw as porosity induced by balling.

### 3.2. Build Rate Influence on Defects

**Figure 7:**
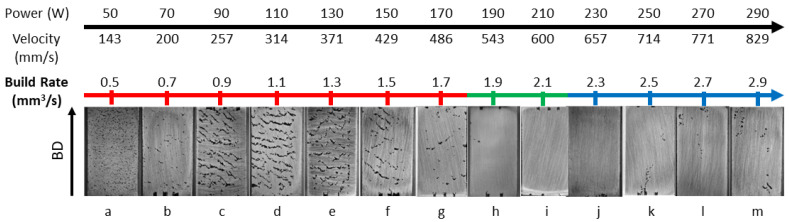
Variation in build rate and corresponding defects across printed samples (constant hatch spacing series, H = 70 μm). Defects change with build rate: low rates (0.5–1.7 mm^3^/s) show lack of fusion, intermediate rates (1.9–2.1 mm^3^/s) are near-dense, and high rates (2.3–2.9 mm^3^/s) show porosity due to balling.

The build rate is the volumetric material deposition rate during the printing process. It is calculated using Equation (2). This equation takes as inputs the laser scan speed, hatch spacing, and layer thickness to determine the volumetric rate of material deposition. For all 13 samples, the hatch spacing and layer thickness maintained constant. Only the variation in the laser scan speed caused the variation in the build rate. From the optical microscopic images of all the samples (see Figure 7), it was observed that, with the increase in build rate, there was a great deal of variation in the cross-sectional structure and observed defect type. The samples that were printed with a build rate of 0.5–1.7 ${\mathrm{mm}}^{3}/s$ suffered from a lack of fusion defect. Samples that were printed with a build rate of 1.9–2.1 ${\mathrm{mm}}^{3}/s$ showed near-dense defect structure. Finally, samples that were printed with a build rate of 2.3–2.9 ${\mathrm{mm}}^{3}/s$ showed porosity defects attributed to balling. This indicates that small variations in the build rate can cause variations in the defect structures.

**Figure 8:**
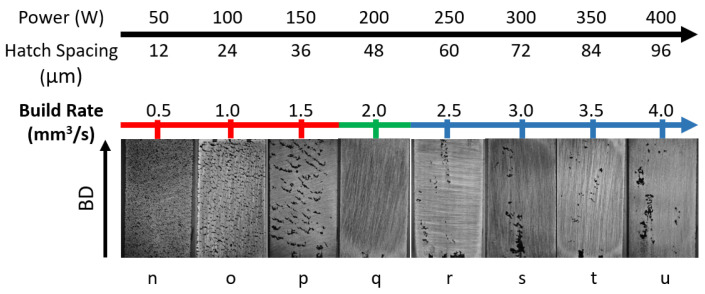
Variation in the build rate and corresponding defects across printed samples (constant velocity series, V = 829 mm/s). Defects change with build rate: low rates (0.5–1.5 mm^3^/s) show lack of fusion, intermediate rates (2.0 mm^3^/s) are near-dense, and high rates (2.5–4.0 mm^3^/s) show porosity due to balling.

To further validate that minor variations in build rate can significantly impact the density of the printed samples, another batch of samples from a series with constant velocity (see Figure 8) was analyzed. These samples were printed using V = 829 mm/s and variable power and hatch spacing, as shown in Figure 8 while maintaining an optimal VED of 100 J/mm^3^ and a layer thickness of L = 50 μm. The analysis shows a trend similar to previous observations. At lower build rates, ranging from 0.5 to 1.5 mm^3^/s, samples suffer from LOF defects. At a higher build rate of 2.5 to 4 mm^3^/s, defects associated with balling appear. In between the LOF and balling cases, the highest densification of the samples is observed. This suggests that, even with an optimal VED, slight variations in the build rate as small as 0.2 to 0.5 mm^3^/s can significantly affect the defect types in printed parts, ultimately impacting their densities. Therefore, optimizing the build rate and process parameters is crucial to achieving the highest density and minimizing the defects in LPBF-printed components.

## 4. Conclusions

A comprehensive defect analysis, using X-ray computed tomography data, was conducted on the laser powder bed fusion (LPBF) SS316L material, which was printed under conditions of an optimal VED of 100 J/mm^3^. This study demonstrates that, despite maintaining an optimal VED and a constant hatch spacing, variations in key process parameters (power and velocity) lead to differences in defect formation and build rate within printed parts. Samples printed at lower power–velocity combinations resulted in a reduced build rate, ranging from 0.5–1.7$\;{\mathrm{mm}}^{3}/s$, and they exhibited defects associated with lack of fusion (LOF). These samples had densities ranging from 90.36–98.8%, with one sample showing the lowest density and another exhibiting the largest Feret diameter of all the printed samples. In contrast, samples printed with a build rate of 1.9–2.1 ${\mathrm{mm}}^{3}/s$ exhibited the highest density, reaching near-full density of ≥99.75%. These samples had optimized process parameters, which resulted in a reduction in defects, thus increasing density. The samples printed at high power–velocity combinations with a build rate of 2.3–2.9 ${\mathrm{mm}}^{3}/s$ exhibited defects associated with the formation of undesirable humps and balls, which is commonly referred to as balling. These samples showed a decrease in density ranging from 98.06–99.63% when compared to the near-dense samples. The density decreased as the Feret diameter started to increase in the samples from 8.09–13.73 mm. The variability in defect types, based on process parameters, suggests that defects can still occur even under optimal VED conditions.

The changes in the defect category based on the build rate indicate that a small change in the build rate can cause a significant variation in the defects present in the printed components. To confirm this finding, a second set of samples printed with a constant velocity and variable power and hatch spacing, at the same VED value, was analyzed. Surface imaging data analysis showed that a build rate of 0.5–1.5 ${\mathrm{mm}}^{3}/s$ causes LOF defect formation, a build rate of $2.0\;{\mathrm{mm}}^{3}/s$ results in a near fully-dense sample, and build rate values of 2.5–4.0 ${\mathrm{mm}}^{3}/s$ result in samples that exhibit balling defects. The second set of samples allowed us to confirm that the build rate effects on densification in the constant velocity series followed a similar trend as the constant hatch spacing series. A value of build rate around $2\;{\mathrm{mm}}^{3}/s$ is recommended while fabricating SS316L at a VED value of 100 J/mm^3^ when using a LPBF system that is equipped with a beam diameter of 125 μm. This highlights the necessity of precise control of process parameters and build rate in the power–velocity (P-V) space to achieve dense, defect-free parts. Additionally, it was observed that fiducial marking can serve as a source of defect generation. Due to the change in the underlying structure in the marking zone, defects instantiate from this region. Therefore, to avoid any unintentional imperfection in the printed parts, it is recommended to limit, and avoid when possible, the design of overhanging geometrical features or fiducial marking in the samples.

## Figures and Tables

**Table 1:** Process parameters for the LPBF SS316L printed at a constant VED of 100 J/mm^3^.

Sample No	Laser Power (W)	Laser Velocity (mm/s)	Build Rate (mm^3^/s)	Volume Density * %
a	50	143	0.5	94.71
b	70	200	0.7	98.80
c	90	257	0.9	92.57
d	110	314	1.1	90.36
e	130	371	1.3	91.17
f	150	429	1.5	96.61
g	170	486	1.7	97.81
h	190	543	1.9	99.88
i	210	600	2.1	99.75
j	230	657	2.3	99.63
k	250	714	2.5	99.38
l	270	771	2.7	98.41
m	290	829	2.9	98.06

* Calculated based on X-ray computed tomography (XCT) analysis.

## Data Availability

The raw data supporting the conclusions of this article will be made available by the authors on request.

## Abbreviations

The following abbreviations are used in this manuscript:

LPBF	Laser Powder Bed Fusion
VED	Volumetric Energy Density
AM	Additive Manufacturing
LOF	Lack of Fusion
OM	Optical Microscopy
XCT	X-ray Computed Tomography
EDM	Electrical Discharge Machining
CT	Computed Tomography

## References

[1] Conner B.P., Manogharan G.P., Martof A.N., Rodomsky L.M., Rodomsky C.M., Jordan D.C., Limperos J.W. (2014). Making sense of 3-D printing: Creating a map of additive manufacturing products and services. Addit. Manuf..

[2] Mostafaei A., Zhao C., He Y., Ghiaasiaan S.R., Shi B., Shao S., Shamsaei N., Wu Z., Kouraytem N., Sun T. (2022). Defects and anomalies in powder bed fusion metal additive manufacturing. Curr. Opin. Solid State Mater. Sci..

[3] DebRoy T., Wei H., Zuback J., Mukherjee T., Elmer J., Milewski J., Beese A.M., Wilson-Heid A.d., De A., Zhang W. (2018). Additive manufacturing of metallic components—Process, structure and properties. Prog. Mater. Sci..

[4] Gordon J.V., Narra S.P., Cunningham R.W., Liu H., Chen H., Suter R.M., Beuth J.L., Rollett A.D. (2020). Defect structure process maps for laser powder bed fusion additive manufacturing. Addit. Manuf..

[5] Whip B.R. (2018). Effect of Process Parameters on the Surface Roughness and Mechanical Performance of Additively Manufactured Alloy 718. Master’s Thesis.

[6] Young Z.A., Coday M.M., Guo Q., Qu M., Hojjatzadeh S.M.H., Escano L.I., Fezzaa K., Sun T., Chen L. (2022). Uncertainties induced by processing parameter variation in selective laser melting of Ti6Al4V revealed by in-situ X-ray imaging. Materials.

[7] Ghouse S., Babu S., Van Arkel R.J., Nai K., Hooper P.A., Jeffers J.R. (2017). The influence of laser parameters and scanning strategies on the mechanical properties of a stochastic porous material. Mater. Des..

[8] Brika S.E., Letenneur M., Dion C.A., Brailovski V. (2020). Influence of particle morphology and size distribution on the powder flowability and laser powder bed fusion manufacturability of Ti-6Al-4V alloy. Addit. Manuf..

[9] Sanaei N., Fatemi A. (2021). Defects in additive manufactured metals and their effect on fatigue performance: A state-of-the-art review. Prog. Mater. Sci..

[10] Zhang B., Li Y., Bai Q. (2017). Defect formation mechanisms in selective laser melting: A review. Chin. J. Mech. Eng..

[11] Tang M., Pistorius P.C., Beuth J.L. (2017). Prediction of lack-of-fusion porosity for powder bed fusion. Addit. Manuf..

[12] Hastie J.C., Kartal M.E., Carter L.N., Attallah M.M., Mulvihill D.M. (2020). Classifying shape of internal pores within AlSi10Mg alloy manufactured by laser powder bed fusion using 3D X-ray micro computed tomography: Influence of processing parameters and heat treatment. Mater. Charact..

[13] Seifi M., Salem A., Satko D., Shaffer J., Lewandowski J.J. (2017). Defect distribution and microstructure heterogeneity effects on fracture resistance and fatigue behavior of EBM Ti–6Al–4V. Int. J. Fatigue.

[14] Åkerfeldt P., Antti M.L., Pederson R. (2016). Influence of microstructure on mechanical properties of laser metal wire-deposited Ti-6Al-4V. Mater. Sci. Eng. A.

[15] Forien J.B., Calta N.P., DePond P.J., Guss G.M., Roehling T.T., Matthews M.J. (2020). Detecting keyhole pore defects and monitoring process signatures during laser powder bed fusion: A correlation between in situ pyrometry and ex situ X-ray radiography. Addit. Manuf..

[16] Qu M., Guo Q., Escano L.I., Clark S.J., Fezzaa K., Chen L. (2022). Mitigating keyhole pore formation by nanoparticles during laser powder bed fusion additive manufacturing. Addit. Manuf. Lett..

[17] Kouraytem N., Li X., Cunningham R., Zhao C., Parab N., Sun T., Rollett A.D., Spear A.D., Tan W. (2019). Effect of laser-matter interaction on molten pool flow and keyhole dynamics. Phys. Rev. Appl..

[18] Cunningham R.W. (2018). Defect Formation Mechanisms in Powder-Bed Metal Additive Manufacturing. P.hD. Thesis.

[19] Guo C., Li S., Shi S., Li X., Hu X., Zhu Q., Ward R.M. (2020). Effect of processing parameters on surface roughness, porosity and cracking of as-built IN738LC parts fabricated by laser powder bed fusion. J. Mater. Process. Technol..

[20] Kumar A., DebRoy T. (2006). Toward a unified model to prevent humping defects in gas tungsten arc welding. Weld. J.-N. Y.-.

[21] Parida R.P., Senthilkumar V. (2021). Experimental studies of defect generation in selective laser melted Inconel 718 alloy. Mater. Today Proc..

[22] Li R., Liu J., Shi Y., Wang L., Jiang W. (2012). Balling behavior of stainless steel and nickel powder during selective laser melting process. Int. J. Adv. Manuf. Technol..

[23] Bertoli U.S., Wolfer A.J., Matthews M.J., Delplanque J.P.R., Schoenung J.M. (2017). On the limitations of volumetric energy density as a design parameter for selective laser melting. Mater. Des..

[24] Darvish K., Chen Z., Pasang T. (2016). Reducing lack of fusion during selective laser melting of CoCrMo alloy: Effect of laser power on geometrical features of tracks. Mater. Des..

[25] Gong H., Rafi K., Gu H., Starr T., Stucker B. (2014). Analysis of defect generation in Ti–6Al–4V parts made using powder bed fusion additive manufacturing processes. Addit. Manuf..

[26] Tucho W.M., Lysne V.H., Austbø H., Sjolyst-Kverneland A., Hansen V. (2018). Investigation of effects of process parameters on microstructure and hardness of SLM manufactured SS316L. J. Alloy. Compd..

[27] de Leon Nope G., Perez-Andrade L., Corona-Castuera J., Espinosa-Arbelaez D., Muñoz-Saldaña J., Alvarado-Orozco J. (2021). Study of volumetric energy density limitations on the IN718 mesostructure and microstructure in laser powder bed fusion process. J. Manuf. Process..

[28] Cherry J., Davies H., Mehmood S., Lavery N., Brown S., Sienz J. (2015). Investigation into the effect of process parameters on microstructural and physical properties of 316L stainless steel parts by selective laser melting. Int. J. Adv. Manuf. Technol..

[29] Eliasu A., Czekanski A., Boakye-Yiadom S. (2021). Effect of laser powder bed fusion parameters on the microstructural evolution and hardness of 316L stainless steel. Int. J. Adv. Manuf. Technol..

[30] Diaz Vallejo N., Lucas C., Ayers N., Graydon K., Hyer H., Sohn Y. (2021). Process optimization and microstructure analysis to understand laser powder bed fusion of 316l stainless steel. Metals.

[31] Byun T.S., Collins D.A., Le Coq A.G., Lach T.G., Linton K.D., Gussev M.N., Werden J.W., Mcalister M.R., Chen X., Joslin C.B. (2021). Mechanical Properties of Additively Manufactured 316L Stainless Steel Before and After Neutron Irradiation (FY21).

[32] Kruth J.P., Bartscher M., Carmignato S., Schmitt R., De Chiffre L., Weckenmann A. (2011). Computed tomography for dimensional metrology. CIRP Ann..

[33] (2022). Dragonfly 2022.2.

[34] Tammas-Williams S., Zhao H., Léonard F., Derguti F., Todd I., Prangnell P.B. (2015). XCT analysis of the influence of melt strategies on defect population in Ti–6Al–4V components manufactured by Selective Electron Beam Melting. Mater. Charact..

[35] Mukherjee T., Zuback J., De A., DebRoy T. (2016). Printability of alloys for additive manufacturing. Sci. Rep..

[36] Rosenthal D. (1941). Mathematical theory of heat distribution during welding and cutting. Weld. J..

[37] Patel S., Vlasea M. (2020). Melting modes in laser powder bed fusion. Materialia.

[38] Masmoudi A., Bolot R., Coddet C. (2015). Investigation of the laser–powder–atmosphere interaction zone during the selective laser melting process. J. Mater. Process. Technol..

[39] Khairallah S.A., Anderson A. (2014). Mesoscopic simulation model of selective laser melting of stainless steel powder. J. Mater. Process. Technol..

[40] King W.E., Barth H.D., Castillo V.M., Gallegos G.F., Gibbs J.W., Hahn D.E., Kamath C., Rubenchik A.M. (2014). Observation of keyhole-mode laser melting in laser powder-bed fusion additive manufacturing. J. Mater. Process. Technol..

[41] Guo Q., Zhao C., Qu M., Xiong L., Escano L.I., Hojjatzadeh S.M.H., Parab N.D., Fezzaa K., Everhart W., Sun T. (2019). In-situ characterization and quantification of melt pool variation under constant input energy density in laser powder bed fusion additive manufacturing process. Addit. Manuf..

[42] Reijonen J., Revuelta A., Riipinen T., Ruusuvuori K., Puukko P. (2020). On the effect of shielding gas flow on porosity and melt pool geometry in laser powder bed fusion additive manufacturing. Addit. Manuf..

[43] Trapp J., Rubenchik A.M., Guss G., Matthews M.J. (2017). In situ absorptivity measurements of metallic powders during laser powder-bed fusion additive manufacturing. Appl. Mater. Today.

[44] Gunenthiram V., Peyre P., Schneider M., Dal M., Coste F., Fabbro R. (2017). Analysis of laser–melt pool–powder bed interaction during the selective laser melting of a stainless steel. J. Laser Appl..

